#  Systematic Differences in HIV, Syphilis and Risk Behaviors among Street Based and Establishment Based Female Sex Workers in Kathmandu Valley of Nepal 

**DOI:** 10.3126/nje.v6i4.17256

**Published:** 2016-12-31

**Authors:** Sampurna Kakchapati, Tarun Paudel, Manju Maharjan, Apiradee Lim

**Affiliations:** 1 Post-doctoral Research Scientist, Department of Computer Science and Mathematics, Faculty of Science and Technology Prince of Songkla University, Pattani, Thailand.; 2 Director, National Centre for AIDS and STD Control, Kathmandu Nepal; 3 Research Officer, CREHPA, Kathmandu Nepal; 4 Assistant Professor, Program in Research Methodology, Department of Computer Science and Mathematics Prince of Songkla University, Pattani, Thailand.

**Keywords:** HIV, Syphilis, Risk behavior, Street, Establishment, Nepal

## Abstract

**Background:**

Female Sex Workers (FSWs) are main drivers of the HIV epidemic in Nepal. The work environment of sex work in Nepal is differentiated into establishment based (e.g. massage parlors, dance restaurants, hotels and lodges) and street based (e.g. streets, parks and markets). The study compares HIV, syphilis and risk behaviours among establishment-based FSWs and street-based FSWs in Kathmandu Valley of Nepal.

**Materials and Methods:**

Cross-sectional bio-behavioral surveys in 2006, 2008, 2011 and 2015 aimed to sample 2093 FSWs using two stage cluster sampling in the Kathmandu valley. Statistical analysis used chi-squared tests and logistic regression models to assess differences of HIV, syphilis and risk behaviors among street-based FSWs and establishment-based FSWs.

**Results:**

The study included 39.7% street-based FSWs and 60.3% establishment-based FSWs. The street-based FSWs had lower education levels, older age groups, separated, longer duration of sex work and inconsistent condom used with clients than establishment-based FSWs (p<0.05). Establishment-based FSWs were lower exposure to HIV intervention programs and pervasive alcohol consumption and use of drugs (p<0.05). The multivariate analysis showed that street-based FSWs were more likely of HIV test (aOR=1.25, 95%CI=1.04, 1.49), HIV (aOR=4.72, 95%CI=2.19, 10.15) and syphilis (aOR=7.96, 95%CI=3.49, 18.15) than establishment-based FSWs.

**Conclusion:**

Street-based FSWs possessed higher risk behaviour and have higher HIV and syphilis prevalence. HIV prevention interventions targeting FSWs should consider risks and vulnerability of street-based FSWs. .

## Introduction

Compared to the general female population, Female Sex Workers (FSWs) are 13.5 times more likely to be infected with HIV [ 1 ]. Globally, FSWs are a high-risk group susceptible to acquiring HIV and sexually transmitted infections (STIs) as well as transmitting the virus to others [ 2 ]. FSWs in Nepal differ by different geographical setting and more confined in the urban cities such as Kathmandu and Pokhara and Terai highway districts [ 3, 7 ]. Kathmandu Valley had highest concentration of FSWs in the country estimated from 10,457 to 11,653 in 2011 [ 4 ]. Many young girls get entered into sex work migrating from rural areas to Kathmandu Valley in quest of work. In the urban cities, they are exposed to situations, making then expose to risky sexual behaviors. Being young and new to the profession, FSWs tend to serve a higher number of clients and have a higher number of working days and possess high risk sexual behaviors. Moreover, FSWs in Kathmandu Valley are often from the lower castes, young age, establishment based and are poor, uneducated, and came from rural areas [ 5 ]. Because of limited education, poor socio-economic status, new to profession, young age and poor knowledge of HIV/AIDS, these groups of FSWs are highly vulnerable for HIV and STI infection [ 8, 10 ]. 

 FSWs in Kathmandu Valley are categorized into street-based and establishment based where FSWs solicit their clients [ 6 ]. The establishment-based FSWs are based on cabin restaurants, restaurants, massage parlors, hotel where they find their clients. Street-based FSWs worked on streets, markets and roads to solicit their clients. Studies and IBBS surveys have investigated HIV, STI and HIV risk behaviors among FSWs in Nepal, such as unprotected sex work and injecting drugs in relation to category of sex work [ 6, 8, 10 ]. It was found that establishment-based FSWs are lower HIV and STI prevalence than street-based FSWs [ 6, 8, 10 ]. Street-based FSWs have a large number of clients, are less likely to use condoms, charge low for sex, and are involved in sex work for a greater number of years than establishment-based FSWs [ 8, 9, 11, 12 ]. Consequently, the vulnerability of street-based FSWs to HIV/STIs is much higher than establishment-based FSWs. Street-based FSWs are also more potential to inject drugs, lacked of consistent condom used and have sex with clients who are drug users, transport workers [ 6, 8, 11 ]. Moreover, street-based FSWs in highway districts have the history of unprotected sexual contact with transportation workers and with migrants who travel from adjacent districts to abroad [ 5, 6 ].

Understanding HIV related risk behavior between these groups of FSWs is of great importance for prevention. Therefore, HIV risk behaviors among FSWs must be examined in relation to their specific work venues or environments. Moreover, this study also compare the structural risk factors (HIV and STI prevention and treatment services) among street-based FSWs and establishment-based FSWs that have not been analyzed before in Nepal.

## Methodology

### Study design and the participants:

A retrospective analysis of Integrated Biological and Behavioral Surveillance of Kathmandu Valley from 2006 to 2015 was conducted. IBBS surveys were cross-sectional in design. FSWs were classified into establishment-based FSWs and street-based FSWs based on their work venues (the places where they solicited or met their clients). The establishment-based FSWs solicited their clients from hotels, lodges, restaurants, massage parlors and guest houses whereas street-based FSWs find their clients from the street, roads, squatter settlements and bhattipasals.

### Sample size calculation

For IBBS Survey, sample size was determined by applying the formula 



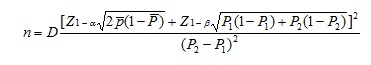



Where n was required minimum sample size per survey, D was design effect (default value of 2), P1 was the estimated number of an indicator measured as a proportion at the time of the first survey, P2 was the expected level of the indicator either at some future date such that the quantity (P2-P1) is the size of the magnitude of change it is desired to be able to detect, Zα was the Z-score corresponding to the degree of confidence with which it is desired to be able to conclude that an observed change of size (P2-P1) would not have occurred by chance (α– the level of statistical significance) and Zβ was the Z-score corresponding to the degree of confidence with which it is desired to be certain of detecting a change of size (P1-P2) if one actually occurred (β– statistical power).

### Data Collection

Data collection consisted of both biological and behavioral data, including handling of biological data for external quality assurance. IBBS survey used a structured questionnaire to assess background characteristics, sexual risk behaviors, usage of condoms, knowledge and awareness of HIV/AIDS and STIs, violence, exposure to HIV/AIDS programs and drug injecting behaviors, 

### Inclusion criteria:

Inclusion criteria was FSWs aged 16 years and above who reported being paid in cash or kind for sex with a male within the last six months

### Exclusion criteria:

Exclusion criteria were FSWs aged 15 years and who didn’t provide consent for the survey. 

### Outcome Variable:

HIV, syphilis and risk behaviours, structural risk factors comparing street-based FSWs and establishment-based FSWs was the outcome variable of the study. 

### Explanatory Variable

The independent variables for the study were background characteristics (year, region, education, marital status), sexual behaviors (duration of sex work, age at first sexual contact, average number of the clients, number of working days), consistent condom use with different sex partners and injecting behaviors (the use of drugs, injecting practices), alcohol use, comprehensive knowledge on HIV (ABC, BCDEF) and Exposure to HIV prevention program (HIV test, Met with Outreach Educators/Peer Educators/Community Mobilizer, Visited Drop In Center (DIC), Visited Sexually Transmitted Infection (STI) clinic and Visited HIV Counseling and Testing (HCT) center). Knowledge on ABC was defined to be aware of A (abstinence from sex), B (being faithful to one partner), and C (consistent and correct condom use or use of a condom during every sex act) as HIV preventive measures respectively. Additionally, DEF refer to knowledge that: a healthy-looking person can be infected with HIV (D), a person cannot get HIV from a mosquito bite (E), and one cannot get HIV by sharing a meal with an HIV-infected person (F). The sex partners of the FSW were categorized as clients, regular clients, non-paying partners, and other partners. Clients and regular partners are those partners who pay for sexual contact. Non-paying partners comprised boyfriends, husbands, intimate partners or those who do not pay for sexual services. Partners other than clients, husbands, and male friend(s) were categorized as other partners which comprises of include massage owners and police officials. Other partners mostly don’t pay for sexual contact with FSWs.

### Ethical Committee Approval

Ethical approval for IBBS surveys were permitted from Nepal Health Research Council. The NHRC approval number for the survey was 1232. Informed consent was obtained from FSWs before the interview. There may be a risk of identifying the FSWs through their signatures if written consent was used. The informed consent was read in the mien of a witness (community motivators or another member of the study team) who then signed the consent form. Study centers with laboratories/clinic were set up at easily accessible locations and individual interviews, clinical examinations and blood collection were carried out in separate rooms. Blood samples were collected from FSWs and were tested for HIV and syphilis. 

### Data Management and Statistical Analysis

Chi-square test was performed to assess FSW's demographic information, sexual behaviours, injecting behaviours, HIV knowledge, exposure to HIV intervention programs, HIV and syphilis, by their work venues (Street and Establishment). Multivariable logistic regression models was used to examine the association of street-based FSWs and establishment-based FSWs with HIV risk, HIV and syphilis while controlling variables that were significant in the bivariate analyses. A p-value of less than 0.05 was used to determine statistical significance. Adjusted odd radios (aOR) as well as their 95% confidence intervals (95% CI) were used to depict the independent relationship between predictors and dependent variables. R program was used for statistical analysis.

## Results

A total of 2093 FSW were included in the analysis from 2006 to 2015, of whom 39.7% of FSWs (230) were street-based FSWs and 60.3% of FSWs (1261) were establishment-based FSWs. There were significant difference of age, education, marital status and duration of sex work between street-based FSWs and establishment-based FSWs, as shown in Table 1. Establishment-based FSWs (82%) were significantly younger than street-based FSWs (59%). Street-based FSWs were significantly less educated than establishment-based FSWs, as 44% of street-based FSWs had no education whereas 23.5% of establishment-based FSWs had no education. There were significant group differences in marital status; establishment-based FSWs (28%) were likely to be single than street-based FSWs (14%). Moreover, nearly one third of street-based FSWs were separated. Street-based FSWs (30.2%) who have worked more than three years was significantly higher than establishment-based FSWs (16.4%).

Table 2 indicates that the ages at sexual debut and consistent condom use with client were signiﬁcantly difference between the establishment-based FSWs and street-based FSWs. Establishment-based FSWs (93.7%) below 20 years were significantly had their first sexual contact compared to 91% among street-based FSWs. Street-based FSWs (9%) had significantly lower consistent condom use with clients than establishment-based FSWs (6%). There were significant difference in used of drugs and alcohol consumption among street-based and establishment-based FSWs. Establishment-based FSWs consumed more alcohol (7.9% versus 5.5%) and used drugs (74.6% versus 68.1%) than street-based FSWs. Less than one percent of FSWs injected drugs and there was no significant difference of injecting behaviors between two groups. HIV test, exposure to DIC, STI and HCT were significantly difference between street-based and establishment-based FSWs, as shown in Table 3. It was found that exposure to HIV intervention programs was low among establishment-based FSWs than street-based FSWs. Compared to establishment-based FSWs, street-based FSWs had higher HIV test (57% versus 51%), visited DIC (40.4% versus 31.8%), STI (34.6% versus 30.5%) and HCT (42.4% versus 31%) than their counterpart in the past year. 

Table 4 shows the comparison of HIV, syphilis among street-based and establishment-based FSWs in Kathmandu Valley. There were significant difference HIV and syphilis among street-based and establishment based FSWs. About 4% street-based FSWs were HIV positive whereas fewer (0.7%) have HIV positive among establishment-based FSWs. In addition to this, street- based had higher syphilis (4.2%) than establishment based FSWs (0.6%).

Table 5 shows the multivariate analyses for HIV risk among street-based FSWs and establishment-based FSWs in Kathmandu Valley. Compared to establishment-based FSWs, street-based FSWs were more likely to have an HIV testing (aOR=1.25, 95%CI=1.04, 1.49), HIV (aOR=4.72, 95%CI=2.19, 10.15) and syphilis (aOR=7.96, 95%CI=3.49, 18.15). However, street-based FSWs are less likely to consumed alcohol (aOR=0.69, 95%CI=0.57, 0.84) than establishment-based FSWs. 

## Discussion

This study compared HIV risk behaviors among FSWs in Kathmandu Valley of Nepal who engage in commercial sex with male clients through different distinct venues (street and establishment). Moreover this study also analyzed structural risk factors (HIV and STI prevention and treatment services) between two groups for first time.

###  Comparison of socio-demographic characteristics, sexual behaviors, drug injecting behaviors, HIV and Syphilis between street-based FSWs and establishment-based FSWs 

#### Age

 Older age was associated among street-based and establishment based FSWs these results corroborate results of other FSW studies showing street-based FSWs are older age groups [ 12, 14 ]. Young FSWs were more likely to base in establishment setting and older FSWs are more likely to base on street for sex work. This may be because of demand of young girls and women in establishment setting due to nature of work and services offer by establishment setting. Furthermore, sex work can be done comparatively more clandestinely and securely in establishment setting. As FSWs get older, they may not be able to continue working in establishment setting because of nature of work they have to do in the establishment setting so they may move out to street to continue sex work.

#### Education

The street-based FSWs had low education than the establishment-based FSWs. Similar to findings from other studies, street based FSWs with less education and knowledge of HIV/AIDS are susceptible for acquiring HIV infection [ 15, 16 ]. Most FSWs are rural areas with low education, making them exposed to risky sexual behaviors and drug involvement. 

#### Sexual behaviors and consistent condom use

The study found that consistent condom use with non-paying partners among FSWs was very low. Overall, 21% among street-based FSWs and 22% among establishment-based FSWs used condom during sex with non-paying partners. Previous IBBS studies of FSWs in Nepal also reported a high prevalence of inconsistent condom use with non-paying partners [ 5, 7 ]. The street-based FSWs were engaged in riskier behaviors when having sex with clients. The proportion of inconsistent condom use with clients during the past week was lower among street-based sex workers compared to establishment-based sex workers. Study identified that street-based FSWs reported not using a condom because of earning extra money [ 11,  16 ]. Besides this, factors such maintaining good relationships with customers, keeping regular customers, avoiding conflicts with or violence from clients also effect the condom use with clients. Moreover, street-based FSWs are also be forced to exchange unpaid and unprotected sex with some law enforcement authorities in order to escape arrest, harassment, obtain release from prison, or not be deported. HIV awareness targeting FSWs alone will not be enough. FSWs are pressured by clients to engage in unprotected sex indicates that the clients are also unaware of the risks for HIV/STI transmission. Clients of FSWs need to be educated about the risks of engaging in unsafe sex with FSWs.

#### Alcohol consumption and injecting behaviors

Alcohol use is common among establishment-based FSWs owing to their work environment. In Nepal at some bars/clubs, employers expect FSWs to encourage customers to drink [ 17 ], alcohol use was part of the routine; that is, drinking with customers at bars/clubs so that customers pay a lot of money. FSWs used alcohol to cope with the stress and violence associated with sex work [ 13, 16, 18 ]. Studies identified that inconsistent condom use with customers among establishment-based FSWs was influenced by their own and their customers' alcohol use [ 8, 18 ]. It is found that establishment-based FSWs had had sex under the influence of alcohol with customers than street FSWs. Moreover, drug use was also prevalent among establishment-based FSWs. Education about alcohol use and its strong influence on unsafe sex must be taught in the HIV prevention education programs for FSWs. Less than one percent of FSWs had injected drugs, and 7% of FSWs used drugs and it was higher among establishment-based FSWs compared to street-based FSWs. Studies found that FSWs were more risk for HIV if they injected drugs [ 8, 15, 16 ]. 

#### HIV and Syphilis

This study found a relatively high prevalence of syphilis among FSWs, although HIV prevalence among FSWs was less than 2%. Both HIV and syphilis were higher among street based FSWs compared to establishment based FSWs consistent with numerous studies indicating high HIV prevalence among street-based FSWs [ 8- 10, 19, 20 ]. 

#### Exposure to HIV intervention programs

The study found that exposure to HIV intervention programs was low among establishment-based FSWs than street-based FSWs. Street-based FSWs were more likely to have HIV test, DIC, STI and HCT visit than their establishment-based FSWs in the past year consistent with the IBBS report [ 6 ]. These findings suggest the need for targeted interventions for the establishment-based FSWs in addition to the general interventions on FSWs. For establishment-based FSWs, FSWs received more advice from their owners/managers for safer sex, access to information and to health services. The major concern of managers/owners of massage parlors was their business and profits rather than the safety or health issues of the FSWs [ 8 ]. Therefore, influence of owners/managers also limits access to HIV intervention services.

## Conclusion

The study found that compared to establishment-based FSWs, street-based FSWs more likely to suffer from HIV and Syphilis. Moreover, street-based FSWs had longer duration of sex work, lacked of consistent condom used with clients compared to establishment-based FSWs. However, establishment-based FSWs are also at high risk because drug and alcohol use is pervasive. Also, exposures to HIV programs are lower in these groups. Therefore, HIV prevention efforts in must target HIV risk behaviors among both FSWs specific to their work environment and social and cognitive factors. Moreover, HIV intervention programs for FSWs must incorporate the social and cultural contexts of sex work and target both FSWs and their clients.

### Limitations of the study

The limitation of the study is IBBS surveys are cross-sectional in design and cannot provide evidence of permit causal inferences. This study was conducted in three regions of Nepal, so the findings will confine to these districts, and may not be generalized to other districts or any other parts of the country. Moreover, there may be possibilities that same FSWs can participate in multiple rounds of surveillance survey because a survey conducted in the same area among the same group over time.

### Future scope of the study:

It is anticipated that this study will help guide policy makers and program managers in identifying useful points and areas to target and focus intervention strategies aimed at different subgroups of FSWs such as street-based FSWs and establishment based FSWs.

### What is already known on this topic:

Prevalence of HIV and Syphilis of FSWs is already known on this topic.

### What this study adds:

Accessing HIV, syphilis and risk behavior between street-based FSWs and establishment-based FSWs is needed for intervention programs. The study suggests important implications for designing surveillance and intervention activities among FSWs based on work venues. This study also compares structural risk factors (HIV and STI prevention and treatment services) between street-based FSWs and establishment-based FSWs that have not been explored before.

## Figures and Tables

**Table 1.** Table 1: Comparison of socio-demographic characteristics between street-based FSWs and establishment-based FSWs

**Characteristics**	**Total** **(n=2093)**	**Street** **(n=832, 39.7%)**	**Establishment** **(n=1261, 60.3%)**	X^2^_(df)_	**P-value**
	**n(%)**	**n(%)**	**n(%)**
**Year**				0.16 (3)	0.98
** 2006 **	500 (23.9)	195 (23.4)	305 (24.2)
** 2008**	500 (23.9)	200 (24)	300 (23.8)
** 2011**	593 (28.3)	237 (28.5)	356 (28.2)
** 2015**	500 (23.9)	200 (24)	300 (23.8)
**Age**				149(2)	<0.001
** Below 20 years**	678 (32.4)	180 (21.6)	498 (39.5)		
** 21-29 years**	840 (40.1)	308 (37)	532 (42.2)		
** 30 years and above**	575 (27.5)	344 (41.3)	231 (18.3)		
**Education**				101(2)	<0.001
** No Education**	660 (31.5)	364 (43.8)	296 (23.5)		
** Primary**	771 (36.8)	274 (32.9)	497 (39.4)		
** Secondary and ** ** Above**	662 (31.6)	194 (23.3)	468 (37.1)		
**Martial Status**				57(2)	<0.001
** Single**	481 (23)	124 (14.9)	357 (28.3)		
** Married**	1059 (50.6)	442 (53.1)	617 (48.9)		
** Separated ** ** (Widow/Divorce)**	553 (26.4)	266 (32)	287 (22.8)		
**Duration of sex work**				60(2)	<0.001
** Less than 1 year**	905 (43.2)	300 (36.1)	605 (48)		
** 1-3 year**	730 (34.9)	281 (33.8)	449 (35.6)		
** More than 3 years **	458 (21.9)	251 (30.2)	207 (16.4)		

**Table:** Table 2: Comparison of sexual behaviors, drug injecting practices between street and establishment

**Characteristics**	**Total** **(n=2093)**	**Street** **(n=832, 39.7%)**	**Establishment** **(n=1261, 60.3%)**	X^2^_(df)_	**P-value**
	**n (%)**	**n (%)**	**n (%)**	
**Age of First Sexual contact**				3.9(1)	0.04
** Below 20 years**	1942 (92.8)	760 (91.3)	1182 (93.7)		
** Above 20 years**	151 (7.2)	72 (8.7)	79 (6.3)	
**Total number of sexual partners**				2.5(1)	0.11
** Less than one**	98 (4.7)	31 (3.7)	67 (5.3)		
** More than one**	1995 (95.3)	801 (96.3)	1194 (94.7)		
**Number of Clients in past week**				3.6(1)	0.056
** Less than one**	147 (7)	47 (5.6)	100 (7.9)		
** More than one**	1946 (93)	785 (94.4)	1161 (92.1)		
**Number of Clients in past year**				2.3(1)	0.12
** Less than one**	1141 (54.5)	436 (52.4)	705 (55.9)		
** More than one**	952 (45.5)	396 (47.6)	556 (44.1)		
**Number of working days in week**				0.03(1)	0.8
** Less than two days**	104 (5)	40 (4.8)	64 (5.1)		
** More than two days**	1989 (95)	792 (95.2)	1197 (94.9)		
**Consistent condom used with Clients**				4.5(1)	0.03
** Yes**	1944 (92.9)	760 (91.3)	1184 (93.9)		
** No**	149 (7.1)	72 (8.7)	77 (6.1)		
**Consistent condom used with regular partners**				0.09(1)	0.7
** Yes**	1483 (70.9)	586 (70.4)	897 (71.1)		
** No**	610 (29.1)	246 (29.6)	364 (28.9)		
**Consistent condom used with Nonpaying partners **				0.39(1)	0.53
** Yes**	451 (21.5)	173 (20.8)	278 (22)		
** No**	1642 (78.5)	659 (79.2)	983 (78)		
**Ever used Drug**				3.9(1)	0.05
** Yes**	145 (6.9)	46 (5.5)	99 (7.9)		
** No**	1948 (93.1)	786 (94.5)	1162 (92.1)		
**Ever Injected Drug**				0.3(1)	0.9
** Yes**	18 (0.9)	8 (1)	10 (0.8)		
** No**	2075 (99.1)	824 (99)	1251 (99.2)		
**Alcohol consumption**				10(1)	0.001
** Yes**	1508 (72)	567 (68.1)	941 (74.6)		
** No**	585 (28)	265 (31.9)	320 (25.4)		

**Table:** Table 3: Comparison of HIV Knowledge, HIV intervention programs

**Characteristics**	**Total** **(n=2093)**	**Street** **(n=832, 39.7%)**	**Establishment** **(n=1261, 60.3%)**	X^2^(_df_)	**P-value**
	**n (%)**	**n (%)**	**n (%)**		
**Ever had HIV test**				5.9(1)	0.01
** Yes **	1123 (53.7)	474 (57)	649 (51.5)		
** No**	970 (46.3)	358 (43)	612 (48.5)		
**Knowledge on ABC**				0.1(1)	0.70
** Yes**	1003 (47.9)	394 (47.4)	609 (48.3)		
** No**	1090 (52.1)	438 (52.6)	652 (51.7)		
**Knowledge on BCDEF**				1.8 (1)	0.17
** Yes**	599 (28.6)	224 (26.9)	375 (29.7)		
** No**	1494 (71.4)	608 (73.1)	886 (70.3)		
**Meet or discussed with OE/PE/CM in past year**				0.03(1)	0.86
** Yes**	1565 (74.8)	620 (74.5)	945 (74.9)		
** No**	528 (25.2)	212 (25.5)	316 (25.1)		
**Visited DIC in past year **				16(1)	<0.001
** Yes**	737 (35.2)	336 (40.4)	401 (31.8)		
** No**	1356 (64.8)	496 (59.6)	860 (68.2)		
**Visited STI clinic in past year **				3.7(1)	0.05
** Yes**	673 (32.2)	288 (34.6)	385 (30.5)		
** No**	1420 (67.8)	544 (65.4)	876 (69.5)		
**Visited HCT in past year**				28(1)	<0.001
** Yes**	744 (35.5)	353 (42.4)	391 (31)		
** No**	1349 (64.5)	479 (57.6)	870 (69)		

**Table:** Table 4: Comparison of HIV, Syphilis between street-based FSWs and establishment-based FSWs

**Characteristics**	**Total** **(n=2093)**	**Street** **(n=832, 39.7%)**	**Establishment** **(n=1261, 60.3%)**	X^2^(_df_)	**P-value**
	**n (%)**	**n (%)**	**n (%)**		
**HIV**				32 (1)	<0.001
** Yes**	38 (1.8)	29 (3.5)	9 (0.7)		
** No**	2055 (98.2)	803 (96.5)	1252 (99.3)		
**Syphilis**				20(1)	<0.001
** Yes**	42 (2)	35 (4.2)	7 (0.6)		
** No**	2051 (98)	797 (95.8)	1254 (99.4)		

**Table:** Table 5: Multivariate analyses for HIV risk between street-based FSWs and establishment-based FSWs * p<.05, **p<.0.01,***p<.0.001

	**HIV risk**		**HIV and Syphilis **	
	**Alcohol Use (aOR, 95%CI)**	**HIV test** **(aOR, 95%CI)**	**HIV** ** (aOR, 95%CI)**	**Syphilis ** **(aOR, 95%CI)**
**Establishment **	1	1	1	1
**Street **	0.69(0.57-0.84)**	1.25(1.04-1.49)*	4.72(2.19-10.15)***	7.96(3.49-18.15)***
